# Urban Growth and urban need to fair distribution of healthcare service: a case study on Shiraz Metropolitan area

**DOI:** 10.1186/s13104-021-05490-2

**Published:** 2021-02-23

**Authors:** Rita Rezaee, Fatemeh Rahimi, Ali Goli

**Affiliations:** 1 Clinical Education Research Center, Health Human Resources Research Center, Shiraz University of Medical Sciences, Shiraz, Iran; 2 Department of Health Information Technology and Management, School of Allied Medical Sciences, Shahid Beheshti University of Medical Sciences, Tehran, Iran; 3 Department of Sociology & Social Planning, Shiraz University, Shiraz, Iran

**Keywords:** Hospital, Location-allocation, Healthcare services, Equity

## Abstract

**Objective:**

(1) To analyze urbanization development pattern in Shiraz after the year 1977; (2) To analyze hospital development model in Shiraz after the year 1977; (3) To review and prioritize location-allocation criteria for hospitals; and (4) To specify appropriate locations for the establishment of potential future general hospitals in Shiraz based on selected criteria.

**Results:**

Although a significant expansion is seen from different geographical directions (particularly northwest and southeast of the city) in the urbanization model after the year 1977, the construction of hospitals has been limited to the central parts of the city and the areas around the city lack any hospitals. The “open access path to the hospital during incidents and disasters and a light traffic” criterion has enjoyed the highest priority amongst the 24 selected hospital location-allocation criteria. Appropriate locations for establishment of new hospitals in the future have been marked as colored maps. The present study has been able to determine and prioritize a comprehensive list of hospital location-allocation criteria. Moreover, the achieved maps from this study can be used by policy makers to develop new hospitals.

## Introduction

According to the World Health Organization (WHO), “the world urban population is expected to grow approximately 1.63% per year between 2020 and 2025, and 1.44% per year between 2025 and 2030” [1]. Rapid and often unplanned urbanization leads to conditions that affect human health in a negative way. Poverty, environmental problems and increasing population demands that outstrip available service capacity are some of these conditions [2]. One of the most important services that can be encountered with deficiency in number and disparity in distribution are hospitals. Therefore, it is necessary to have a precise plan to increase the number of these facilities concurrent with urban population growth. Determining the optimal number and location of hospitals are two of the most important criteria for establishing new ones [3]. Based on our knowledge, various studies have been conducted to select the optimal hospital location; nevertheless, different researchers have used various hospital location criteria in their studies [4–7].

By considering the importance of fair distribution of healthcare services, the present study aims are at the following: (1) To analyze urbanization development pattern in Shiraz after the year 1977; (2) To analyze hospital development trend in Shiraz after the year 1977; (3) To review and prioritize location allocation criteria for hospitals and (4) To specify appropriate locations for the establishment of potential future general hospitals in Shiraz based on selected criteria.

## Main text

### Methodology

At first, Shiraz region land-use maps of 1977, 1987, 2000, and 2016 were extracted from Landsat image. To evaluate the urban growth model, Landsat satellite with 30 m and 60 m resolution was used. The Shiraz land-use map was extracted by supervised classification method and the maximum likelihood algorithm in three classes of land-uses. Meanwhile, the land-use change maps in the city of Shiraz were prepared and the process of urban growth was achieved for Shiraz after the year 1977. Then, the hospital development pattern depicted by Arc GIS 10.3 and the urbanization development model were overlapped.

Then, in order to select an appropriate area for new hospitals based on urban growth, hospital location-allocation indices of the Ministry of Health and Medical Education (HM)^1^ and other studies were investigated. Then, a primary checklist was provided as a questionnaire to 22 experts (Additional file 1: Table S1). Experts team were 10 specialists in Health manager, 8 professionals in urban planning and 4 Community medicine experts. The questionnaires were investigated using the Delphi technique in two rounds (agreement score > 75%).

The achieved criteria were arranged in the form of another questionnaire and compared based on the Analytical hierarchical Process (AHP) by the experts. The results of pairwise comparisons were analyzed by Expert choice software (The inconsistency ratio = 0.1). Questionnaires with a high inconsistency ratio were returned to the experts for revision and correction. Then, the numbers resulting from paired comparisons have entered into the software again, and the absolute weight was calculated.

Finally, the geodatabase of the mentioned criteria was created in Arc GIS. The Weight of every criterion was exerted on its layer. The layers were overlapped and the final raster layer was divided into low, moderate, high, and very high potential areas for the construction of new hospitals.

### Results

**Fig. 1:**
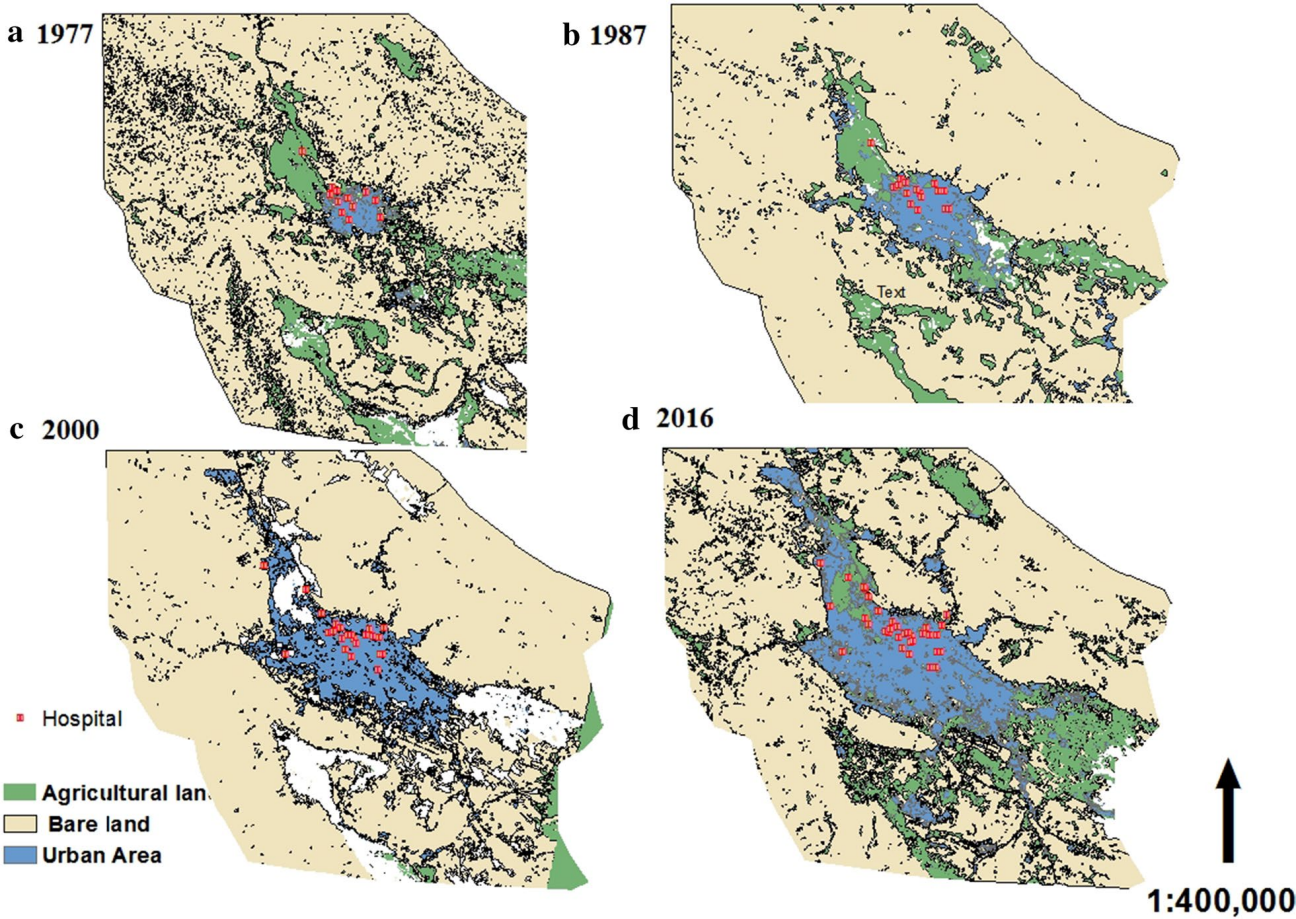
Shiraz Land-use from 1977 until 2016 and hospitals points (The map depicted in figure is our own work)

Shiraz is a provincial-center city, with 11 administrative zones and have 1.609.615 population. In Shiraz, there are 32 hospitals with about 5520 hospitals’ active beds (Additional file 2: Table S2).With the growth in construction, bare lands in the city and surrounding areas have been allocated for the development of the city (Fig. 1).

Although a significant expansion was seen from different geographical directions (particularly in northwest and southeast region) in urban growth after the year 1977, the construction of hospitals has been limited to the central area. Trends of hospitals development since 1977 showed 12 hospitals were built before 1977 (Fig. 1a), 5 hospitals between 1977 and 1987 (Fig. 1b), 7 hospitals between 1987 and 2000 (Fig. 1c), and 10 hospitals after the 2000. The focus of this study is on general hospitals, both public and private. According to the World Health Organization (WHO), more than 1 hospital bed per 1000 people is essential [8]. However, as shown in Additional file 1: Table S1, in terms of the number of general beds (public and private hospitals), except for areas 1, 2, 3, and 6, other areas are completely deprived.

The primary checklist of location-allocation criteria has been provided in Table 1. Following the application of Delphi technique on the primary list, 24 criteria achieved a score of 7.5 from 10. These 24 cases were classified as sub-criteria in 6 main criteria groups (as the main criteria for allocation of location for the hospital). The relative importance of each criterion and its sub-criteria has been given in Table 1.

**Table 1:** The main hospital location-allocation criteria and their relative importance

Main criteria	Weight	Sub-criteria	Weight	Synthetic weight A^a^	Synthetic weight B^b^	Priority
Population	0.1139	Population density	0.3346	0.0381	0.0225	21
		Proximity to the major urban population	0.3915	0.0446	0.0282	19
		pathogenic conditions of the areas	0.2909	0.0331	0.0347	13
Location characteristics	0.1002	Being located in urban areas	0.1723	0.0173	0.0120	24
		Fair distribution all over the city	0.3747	0.0376	0.0301	17
		Fast and easy accessibility	0.4516	0.0453	0.0323	14
Accessibility	0.1977	Proximity to the main streets	0.1858	0.0367	0.0253	20
		Not having heavy traffic in emergency conditions	0.5911	0.1169	0.0863	1
		The possibility of separating the hospital’s main entrance from its emergency entrance	0.2214	0.0438	0.0308	16
Compatibility	0.1696	Being far from the airport	0.0799	0.0136	0.0188	23
		Being far from radio, television, and telecommunication masts	0.0813	0.0138	0.0189	22
		Being far from hills, valleys, and faults	0.1721	0.0292	0.0409	10
		Not being located on the river, landslide, or avalanche path	0.2323	0.0394	0.0557	7
		Being far from industrial centers	0.1158	0.0196	0.0288	18
		Establishment of hospitals in the areas with lower air pollution	0.1591	0.0270	0.0318	15
		Establishment of hospitals in the areas with lower noise pollution	0.1480	0.0251	0.0360	12
Infrastructure	0.2521	Proximity to urban services, such as fire station	0.2451	0.0618	0.0571	6
		Availability of information technology and telecommunication infrastructures	0.1794	0.0452	0.0392	11
		The possibility of establishing facilities, such as parking	0.1847	0.0466	0.0445	8
		Availability of urban infrastructures, such as gas, water, and telephone	0.3908	0.0985	0.0859	2
Land specifications	0.2193	Having adequate area according to HM’s standards	0.2540	0.0557	0.0647	4
		Having a standard geometrical shape	0.1546	0.0339	0.0443	9
		Adapting the land dimensions with HM’s standards	0.2682	0.0588	0.0635	5
		Adapting the width of the roads directed to the hospital with standards	0.3234	0.0709	0.0761	3

^a^Before calculating CR

^b^After calculating CR

**Fig. 2:**
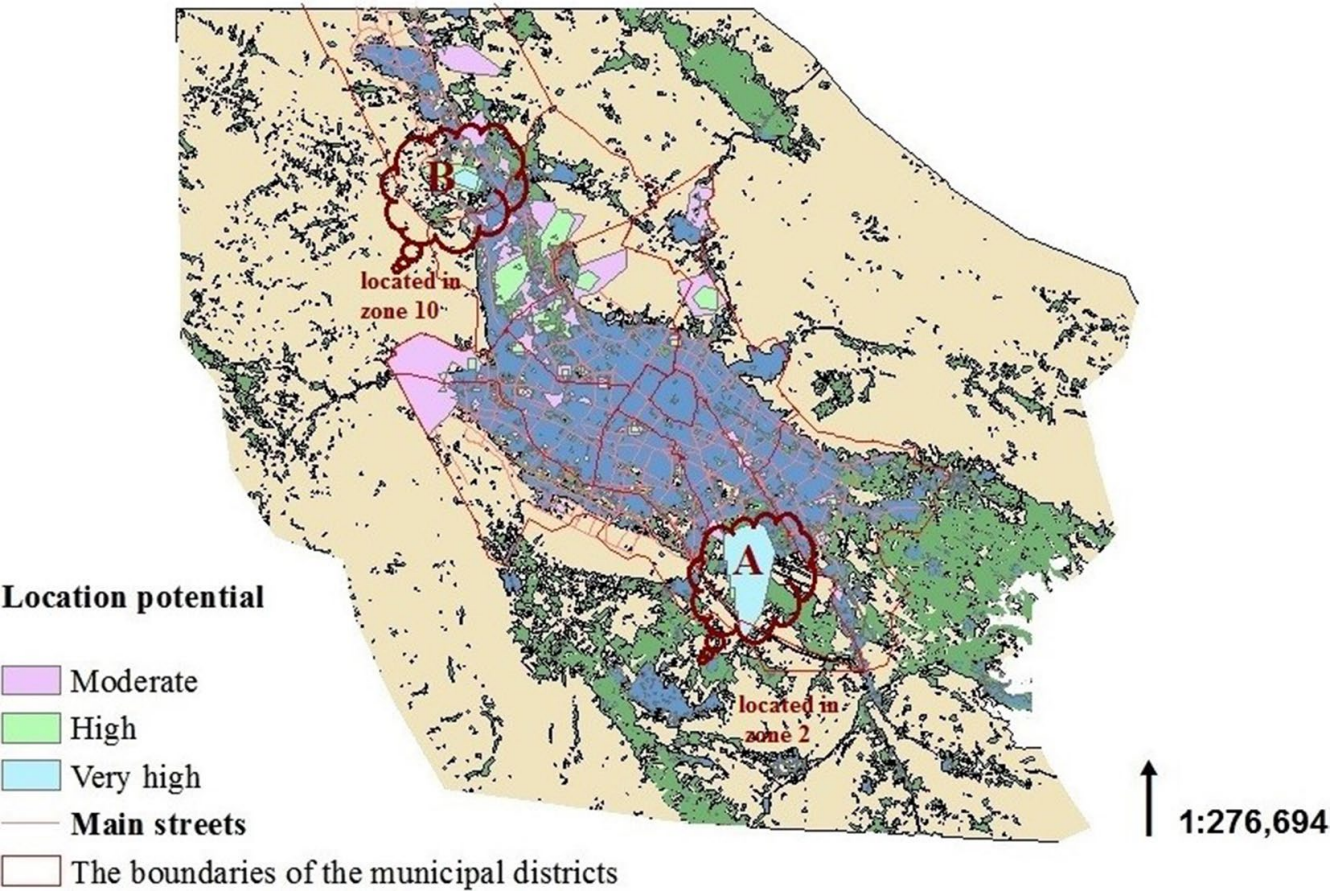
Appropriate locations for establishment of hospitals in the future (The map depicted in figure is our own work)

Appropriate locations for establishment of hospitals in the future have been marked in Fig. 2 with blue color (very high), green (high) and pink (moderate). The specified locations (A and B) have achieved the highest scores. Location A is in the south areas of the city and around municipal zone 2 and location B has a smaller area and located in municipal zone 10 in Shiraz.

Shiraz is the fifth most populous city in Iran [9]. The results of the study showed that since 1977, Shiraz had rapid growth in various geographical directions (particularly the northwest and southeast of the city) (Fig. 1). The results of the study by Movahed (2008) has confirmed that Shiraz has attracted many immigrants from smaller towns and villages around it because of its job opportunities, appropriate facilities and its beauty, and its agricultural and bare lands have decreased from 1921 to 2004, being replaced with a city [10]. Although hospitals have been built concurrent with the development of urbanization, most of them have been intense in the central areas of the city. Considering high demands for healthcare services from Shiraz within south provinces and the countries around the Persian Gulf, and the increasing growth in the number of immigrants, careful planning is needed in the area of hospital location-allocation. It must be noted that hospitals in Shiraz have mostly been constructed on lands endowed by benevolent people. Therefore, in many cases, an appropriate location has not been selected for the construction of hospitals based on standard criteria. Now it is not economically cost-effective to transfer current hospitals to other locations. Since the process of location-allocation has a large effect on increasing accessibility, reducing costs, and launching various activities, this process is considered as one of the most important and effective executive projects [11, 12]. The positive point of the present study is that it has provided a comprehensive checklist of criteria for hospital location-allocation that can be provided for researchers and policymakers in the area of healthcare not only in Shiraz but also in all urban and rural areas.

There are some studies in the existing literature on the use of AHP in hospital location-allocation. Chatterjee and Mukherjee (2013) used three criteria of “geometric shape of the land”, “population density” and “physical development opportunities” to hospital locations in India rural area. Other criteria, same as “land price”, “land ownership”, “distance from educational centers”, and “distance from public transportation stations” identified as effective factors [4]. Sharmin and Neemaee (2013) selected the appropriate location for the construction of a hospital in Dhaka in Bangladesh by AHP. From among the criteria used for this selection, the four criteria of “distance from current hospitals”, “network of passages”, “industrial centers” and “water currents” were identical with the finalized criteria in the present study [13]. Wissem et al. [7] selected the appropriate location for the establishment of a new hospital from three locations suggested in Sfax. From among the criteria used in the study, the criteria of “distance from network of passages”, “population density”, “possibility of providing urban services such as gas”, “distance to other hospitals”, and “air quality” were identical with the finalized criteria in the present study. Although the criteria of “distance to educational centers” and “proximity to public transportation systems” were used in the study, they failed to receive the necessary scores by experts for the city of Shiraz. The results of the study showed that “air quality” was the top priority in allocation of location for hospitals [7]. However, in the present study, “air quality” is in the 15th priority. In general, numerous criteria are effective in selecting an appropriate location for the construction of a hospital. Investigating all these dimensions is not possible through traditional methods. Lack of attention to these criteria in selecting a location can have plenty of damages to financial resources, the environment, people, and urban management [14].

### Conclusion

Considering the views of experts, the standards of the Ministry of Health and Medical Education, and the criteria used in related studies, the present study has been able to determine and prioritize a comprehensive list of criteria for the allocation of an appropriate location for hospitals. On this basis, the criterion of the open-access path to the hospital during incidents and disasters and light traffic has enjoyed the highest priority for the construction of a hospital. Policymakers in the field of health and researchers active in this area can use these criteria as useful scientific tools for selecting the most appropriate locations and investigating the location of existing hospitals. The achieved maps from this study, which have been created based on the determined criteria, can be used by policymakers to develop new hospitals.

## Limitations

No information was found on noise pollution, air pollution, and level of traffic at the time of occurrence of incidents and disasters, and the pathogenic conditions of the areas. Therefore, the final combination of layers was done by disregarding the listed data layers. Data relating to the location of communication and telecommunication towers were not provided for the researchers because of their confidentiality.

## Supplementary Information

**Additional file 1: Table S1.** The primary hospital location-allocation criteria.

**Additional file 2: Table S2.** Characteristics of Shiraz and the hospital beds per Zones.

## Data Availability

Not applicable.

## Abbreviations

WHO: World Health Organization
MH&ME: Ministry of Health and Medical Education
AHP: Analytical hierarchical process
GIS: Geographic information system

## Footnotes

1 AR-0201 guideline.
