# Supplementary material for: Urban Growth and urban need to fair distribution of healthcare service: a case study on Shiraz Metropolitan area
Source: BMC Res Notes. 2021 Feb 23;14:70. doi: 10.1186/s13104-021-05490-2 (PMC7903626; doi:10.1186/s13104-021-05490-2)
Supplement: Supplementary file 1 — Additional file 1: Table S1. The primary hospital location-allocation criteria. [file 13104_2021_5490_MOESM1_ESM.docx]

**Table S1. The primary hospital location-allocation criteria**

| **Criteria** | **Index** |
| --- | --- |
| Location in urban areas | 1 |
| Fair distribution of hospitals all over the city and respecting other hospitals’ service area | 2 |
| Having enough space and being located in the best geographical direction | 3 |
| The possibility to separate the hospital’s entrance from other entrances in case it is located in the university | 4 |
| Having enough space for building houses for specialists (particularly in government hospitals) | 5 |
| The possibility of establishing facilities, such as parking | 6 |
| The possibility of separating the hospital’s main entrance from its emergency entrance | 7 |
| Feasibility of access by helicopter | 8 |
| Proximity to metro | 9 |
| Proximity to the major urban population | 10 |
| Proximity to the main streets | 11 |
| Fast and easy accessibility | 12 |
| Not having heavy traffic in emergency conditions | 13 |
| Availability of urban infrastructures, such as gas, water, and telephone | 14 |
| Availability of information technology and telecommunication infrastructures | 15 |
| Proximity to urban services, such as fire stations | 16 |
| Proximity to parks and green spaces | 17 |
| Being far from military garrison | 18 |
| Being far from airport | 19 |
| Being far from bus, truck, and train terminals | 20 |
| Being far from police station | 21 |
| Being far from radio, television, and telecommunication masts | 22 |
| Being far from educational facilities | 23 |
| Being far from stadiums | 24 |
| Being far from hills, valleys, and faults | 25 |
| Non-proximity of hospital’s main entrance to residential buildings | 26 |
| Not being located on the river, landslide, or avalanche path | 27 |
| No natural barriers, such as fault, and extreme topography through the proposed land | 28 |
| Non-passage of powerful transmission lines, such as high voltage through the proposed land | 29 |
| Being far from industrial centers | 30 |
| Belonging to a legal ownership | 31 |
| No need for destruction of parks and jungles | 32 |
| No need for destruction of residential buildings | 33 |
| Land price | 34 |
| Having adequate area according to the HM’s standards | 35 |
| Having standard geometrical shape | 36 |
| Adapting the land dimensions with the HM’s standards | 37 |
| Adapting the width of the roads directed to the hospital with the HM’s standards | 38 |
| pathogenic conditions of the areas | 39 |
| Being far from the city’s main entrance | 40 |
| Accessibility to other health facilities, such as ultrasound, MRI, and radiology services | 41 |
| The possibility of establishing emergency communication path between hospitals | 42 |
| Establishment of hospitals in the areas with lower air pollution | 43 |
| Establishment of hospitals in the areas with lower noise pollution | 44 |
| Geological, geotechnical, and soil resistance status of the selected land | 45 |
