# Supplementary material for: Urban Growth and urban need to fair distribution of healthcare service: a case study on Shiraz Metropolitan area
Source: BMC Res Notes. 2021 Feb 23;14:70. doi: 10.1186/s13104-021-05490-2 (PMC7903626; doi:10.1186/s13104-021-05490-2)
Supplement: Supplementary file 2 — Additional file 2: Table S2. Characteristics of Shiraz and the hospital beds per Zones. [file 13104_2021_5490_MOESM2_ESM.docx]

**Table S2. Characteristics of the hospital beds per Zones**

| **Zone** | **Population** | **Number of hospitals’ active beds** | | **General beds per 1000 inhabitants** | **Number of hospitals (all types)** |
| --- | --- | --- | --- | --- | --- |
|  |  | **General** | **Specialized** |  |  |
| **1** | 164000 | 2735 | 796 | 17.9 | 17 |
| **2** | 183127 | 467 | 220 | 2.5 | 6 |
| **3** | 144367 | 312 | 497 | 1.6 | 5 |
| **4** | 261663 | 0 | 35 | 0 | 1 |
| **5** | 160497 | 0 | 0 | 0 | 0 |
| **6** | 123000 | 345 | 114 | 3.1 | 3 |
| **7** | 133946 | 0 | 0 | 0 | 0 |
| **8** | 36732 | 0 | 0 | 0 | 0 |
| **9** | 144677 | 0 | 0 | 0 | 0 |
| **10** | 141160 | 0 | 0 | 0 | 0 |
| **11** | 116446 | 0 | 0 | 0 | 0 |
